# High-Throughput Automated Phenotyping of Two Genetic Mouse Models of Huntington's Disease

**DOI:** 10.1371/currents.hd.124aa0d16753f88215776fba102ceb29

**Published:** 2013-07-11

**Authors:** Fuat Balci, Stephen Oakeshott, Jul Lea Shamy, Bassem F. El-Khodor, Igor Filippov, Richard Mushlin, Russell Port, David Connor, Ahmad Paintdakhi, Liliana Menalled, Sylvie Ramboz, David Howland, Seung Kwak, Dani Brunner

**Affiliations:** Department of Psychology, Koç University, Istanbul, Turkey; PsychoGenics Inc., Tarrytown, New York, United States of America; Mount Sinai School of Medicine, New York, New York, USA; Pfizer Inc.; PsychoGenics Inc., Tarrytown, New York, United States of America; PsychoGenics Inc., Tarrytown, New York, United States of America; PsychoGenics Inc., Tarrytown, New York, United States of America; PsychoGenics Inc., Tarrytown, New York, United States of America; PsychoGenics Inc., Tarrytown, New York, United States of America; PsychoGenics Inc., Tarrytown, New York, United States of America; PsychoGenics Inc., Tarrytown, New York, United States of America; CHDI Foundation Inc, Princeton, New Jersey, USA; CHDI Foundation Inc, Princeton, New Jersey, USA; PsychoGenics Inc., Tarrytown, New York, United States of America; Columbia University, New York, New York, USA

## Abstract

Phenotyping with traditional behavioral assays constitutes a major bottleneck in the primary screening, characterization, and validation of genetic mouse models of disease, leading to downstream delays in drug discovery efforts. We present a novel and comprehensive one-stop approach to phenotyping, the PhenoCube™. This system simultaneously captures the cognitive performance, motor activity, and circadian patterns of group-housed mice by use of home-cage operant conditioning modules (IntelliCage) and custom-built computer vision software. We evaluated two different mouse models of Huntington’s Disease (HD), the R6/2 and the BACHD in the PhenoCube™ system. Our results demonstrated that this system can efficiently capture and track alterations in both cognitive performance and locomotor activity patterns associated with these disease models. This work extends our prior demonstration that PhenoCube™ can characterize circadian dysfunction in BACHD mice and shows that this system, with the experimental protocols used, is a sensitive and efficient tool for a first pass high-throughput screening of mouse disease models in general and mouse models of neurodegeneration in particular.

## Introduction

Mouse models of human disease have provided valuable insights into our understanding of human genetic neurological disorders. Generally, first pass validation of animal models is conducted using high throughput assays, in order to rapidly make decisions about which models and compounds to pursue more in-depth. Other more time-consuming assays are a better fit for secondary and tertiary tier testing, which is generally built upon and guided by results of first-order screening and characterization.

Molecular, neuroimaging, neuropathological, and behavioral phenotyping are complementary approaches used together to validate animal models of human disease and to evaluate potential therapeutics. Whereas there are many high-throughput assays for the first three approaches, the options for high-throughput behavioral phenotyping are rather limited, with the result that traditional behavioral tests are still standard practice for first-order validation of animal models. However such testing is frequently slow, labor intensive, difficult to implement and can be vulnerable to subjective factors during experimentation and/or scoring. This traditional approach typically involves a series of sequential tests of one aspect of behavior at a time limiting the ability of these assays to capture the complexity and diversity of the behavioral phenotype of the animals across time. Such tests often require extensive experimenter handling, making it hard to study genetically manipulated animals in non-stressed conditions. It is possible that an ethologically valid environment with less experimenter intervention may be more reflective of the natural state of the animal model and less susceptible to cross-laboratory discrepancies. Recognition of these limitations has led several research laboratories and vendors to focus on automated approaches in behavioral phenotyping: e.g., PhenoMaster (TSE-Systems; 1), IntelliCage 2, HomeCage Scan (Cleversys, Inc.) and “fully-automated live-in environment” 3.

Here we provide a demonstration of our novel high-throughput phenotyping system, PhenoCube^TM^, based on the Intellicage system, using two transgenic models of Huntington’s Disease (HD), the well-characterized R6/2 fragment mouse, and the full length transgenic BACHD mouse developed in the laboratory of X. William Yang 4. By “high-throughput behavioral testing”, we mean efficient and quick screening of animal subjects for multiple phenotypes simultaneously. These phenotypes might include (but are not limited to) cognitive performance, motivation and locomotor, circadian, and exploratory activity. In addition, we showed that the system has high sensitivity and robustness to detect the deficits in these mutant lines.

The R6/2 mouse model expresses a short N-terminal fragment of mutant human huntingtin and is the best-known mouse model of HD 5 in terms of pathophysiology and behavioral abnormalities (e.g., 7 ^,^ 8 ^,^ 9 ^,^ 10 ^,^ 11). R6/2 mice have been shown to be hypoactive (e.g., 12 ^,^ 9), exhibit disrupted circadian patterns (e.g., 13), response acquisition 14 and interval timing 15, and have impaired spatial discrimination and reversal as assessed in water-maze and t-maze tasks (e.g., 8 ^,^ 16 ^,^ 17 ^,^ 18 ^,^ 19). By contrast, the BACHD mouse expresses the full-length human mutant huntingtin gene with 97 glutamine repeats under the control of endogenous htt regulatory machinery on a bacterial artificial chromosome, and has been less widely studied but also has been shown to mimic some aspects of HD pathophysiology and behavioral abnormalities 4 ^,^ 9 ^,^ 20, including circadian abnormalities 14 ^,^ 21.

The PhenoCube^TM^ system acquires a broad range of different measures that span multiple disease-relevant domains (i.e., cognition, locomotor activity and circadian patterns), and thus can efficiently capture the complexity of the behavioral phenotype. We developed the PhenoCube^TM^ through hardware modifications of IntelliCage units and addition of custom-built computer vision hardware and software. This system enables behavioral phenotyping of group-housed mice within a home-cage-like environment over multiple days with minimal experimenter interruption, allowing capture of the endogenous behavioral rhythms of the subjects. Models of HD are particularly relevant disease model for demonstration of the utility of this system as it is characterized by altered cognition, motor activity, and circadian rhythms, all domains that can all be assessed simultaneously by the PhenoCube^TM^. The present experiments capture these behavioral phenotypes using the novel PhenoCube^TM^ apparatus, demonstrating the utility of the system and of the experimental protocols developed in our lab.

## METHODS

**Animals:**

R6/2 (R6/2 CAG 120, CHDI-81001000) transgenic heterozygous and wild type (WT) littermate control mice were obtained from Jackson Laboratories (Bar Harbor,ME; strain B6CBA-Tg[HDexon1]62Gpb/3J, stock number 006494). Mice were generated by crossing ovarian-transplanted females (from R6/2 CBAB6J female donors) with CBAB6F1J WT males. Genotype was determined by polymerase chain reaction (PCR) of tail-tip DNA at 15 days of age 22. CAG repeat lengths were measured by Laragen (Los Angeles, CA, USA) using standard protocols and Genemapper software as previously described 9. R6/2 mice were tested at 7, 8, 9 and 10 weeks of age. The CAG repeat of the R6/2 mutant is 120 + 5.****

BACHD (CHDI-81001012and WT control mice were generated at the Jackson laboratory on an FVB/NJ x C57Bl/6J F1, created by crossing BAC transgenic FVB/NJ males with WT C57Bl/6J females. The congenic BAC FVB/NJ line used for breeding was obtained from the laboratory of X. William Yang at the UCLA David Geffen School of Medicine, Los Angeles. Genotyping was completed by Laragen (Los Angeles, CA, USA). BACHD and the corresponding WT control mice were tested at 16, 24, 36, and 52 weeks of age.

Prior to testing in PhenoCube^TM^, mice were injected with sterile transponders (T-IS 8010 FDX-B, Datamars SA, Bedano, Switzerland) under 2% isoflurane inhalation anesthesia. During recovery from anesthesia, mice were introduced to their social testing groups and housed in genetically homogenous in OptiRAT® cages (Animal Care Systems, CO). The environment was enriched with a play tunnel, shredded paper, and a plastic bone. Food and water were available ad libitum with the exception of water deprivation 16 hours prior to each phase of testing in our system. Temperature and humidity were controlled and monitored daily. This study was carried out in strict accordance with the recommendations in the Guide for the Care and Use of Laboratory Animals, NRC 23. The protocol was approved by the Institutional Animal Care and Use Committee of Psychogenics, Inc. (PHS OLAW animal welfare assurance number A4471-01), an AAALAC International accredited institution (Unit #001213).****

**Apparatus:**

Experiments were conducted using modified Intellicage units (New Behavior, AG, Zurich, CH), each with a Day/Night Camera mounted on top of the cage for Computer Vision (CV) analysis (Figure 1). Intra-maze spatial cues were added to the environment by placing laminated striped paper on the outside of the cage walls, while three climbing structures (two rods, a cubic central object and a three step staircase) were placed inside the cage to provide an enriched topology (Figure 1 – left panel).

The cages were maintained at all times on a 12:12 light/dark cycle, with white light during the day and red light during the night. The light intensity under red light was recorded at 7 lux using a photographic band-pass filter (LDP LLC, NJ) that eliminates long wavelength light frequencies not visible to mice 24 and was sufficient to allow the camera to detect the mice while maintaining a low subjective light level for the mice. The light intensity under white light was 100 lux. Inside the PhenoCube^TM^ environment, water was only available from within the corners while food was freely available on the cage floor at all times. It was sometimes necessary to remove a mouse from the study when no licking was recorded, in order to ensure proper hydration.

**PhenoCube™:**
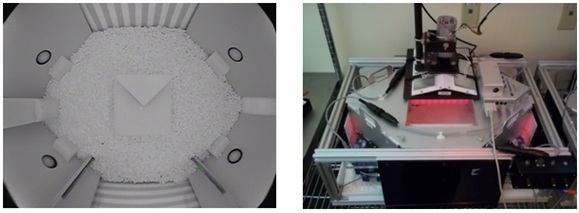
Inside (left panel) and outside view (right panel).

**Procedure:**

*Standard Experimental Procedure:*In all the experiments described here, a standard series of test protocols were employed, defined here as Habituation, Alternation, and Reversal (see Figure 2).

*Habituation: *The Habituation phase (Figure 2 – left panel) was used at the start of the experiment during which mice have access to all four of the corners. As soon as any mouse enters, both doors to the water open and remain open until the mouse leaves the corner. This phase of the experiment constitutes the “magazine training” and allows the mice to learn that water reinforcement is available in the corners. Basic activity data is collected during this phase of the experiments. The overall rate of corner visits and licking provide an insight into the general activity levels of the animals as well as their capacity to obtain water reinforcement, while evaluation of the overall rate of repeated visits (i.e. returning to the previously visited corner) gives some indication of the animals’ general tendency to persevere.

*Alternation: *In the Alternation training protocol (Figure 2 – middle panel), the animals are required to visit two of the four corners in order to gain access to water. For each subject, two adjacent corners (active corners) along one of the shorter sides of the rectangular cage were contingently rewarded, while the other two (exploratory corners) were never rewarded. The Alternation protocol was set up to train the animals to switch between the two active corners, only receiving reinforcement for alternating visits. For example, if corners 1 and 2 were active, an initial visit to corner 1 would be a correct visit and rewarded. To obtain further reinforcement, the mouse was then required to visit corner 2. Repeat visits to corner 1 were classified as incorrect, and mice would not receive a reward there. Following a visit to corner 2, the corners would switch again, such that reinforcement would now be available only in corner 1 and so on. If a mouse incorrectly visits the same active corner more than once or visits an “exploratory” corner after leaving an active corner, the identity of the target correct corner does not change and a visit to that corner will result in available reinforcement; the only event leading to a switch in the correct corner identity is a visit to the currently correct corner, in which reinforcement will be available.

Alternation data was calculated within a 113-s interval of leaving an active corner, such that only a visit to the correct corner 113-s or less after exiting an active corner counted as a correct Alternation, with any visit to the currently *incorrect corner* counting as incorrect, while visits to the *exploratory corners* are irrelevant. Inter visit intervals were cap to minimize the chances that other events interrupted the alternating sequence– the precise interval used is not of great significance, with similar results detected with different intervals. Irrespective of this interval, visits to a correct corner were rewarded – the interval is relevant only to the data handling process. The primary measure employed to assess Alternation performance, percent alternation, is generated by calculating the percentage of visits to active corners made following a visit to either of the two active corners within a particular interval that are correct alternation visits. For example, if corners 1 and 2 are active, the number of visits to corner 2 following a visit to corner 1 plus the number of visits to corner 1 following a visit to corner 2 comprises the total Alternation visits, which are then divided by the total visits to either corner 1 or corner 2. Previous experiments in our laboratories have indicated that, although mice do have some tendency to spontaneously alternate between foraging locations, their alternating behavior within the corners is significantly increased by the type of enforced alternation training employed here, such that performance on this task does appear to be sensitive to the animals’ learning the reinforcement contingencies.

Each corner contained two nosepoke recesses, one on each side, used to deliver water reinforcement during correct visits. Each active corner delivered water only on one side, with the rewarded side dependant on the specific identity of the current corner, such that if only the left side was rewarded in active corner 1, then only the right side was rewarded in active corner 2 and vice versa. When mice made visits to a correct corner (i.e., an alternation visit) and nose-poked on the correct side, the door behind the correct nosepoke recess for that corner opened for 8 s, providing access to water. After 8 s, the door closed, preventing further access to water, with the mice then required to alternate and enter the other active corner for further rewards. No penalty was imposed for initially nosepoking on the incorrect side; the first nosepoke during a correct visit to the correct side always resulted in delivery of reinforcement. However, for the purposes of behavioral analysis, the proportion of initial nosepokes to the correct side was calculated such that, for example, a mouse making a correct visit and subsequently nosepoking to the wrong side and then to the correct side would receive water, but the mouse’s ‘initial nosepoke’ for that visit would be scored as incorrect (from a behavioral efficiency perspective).

*Reversal:* The final phase is a nosepoke reversal (Figure 2 – right panel). The protocol is similar to the Alternation phase, except that the correct nosepoke recesses are switched between the two active corners, such that if the left nosepoke was correct in corner 1, instead the right nosepoke was correct – no differences between the genotypes were observed in performance on this task so these data are not presented, but this information is included here for completeness.

**Graphical depiction of the IntelliCage and the experimental procedures: Habituation, Alternation, and Reversal.**
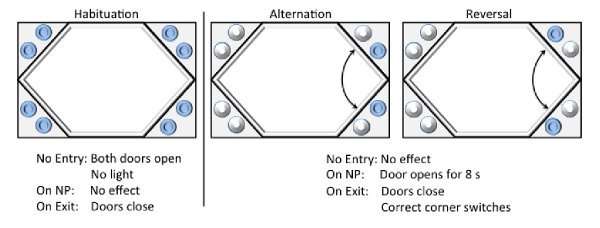
Shaded circles signal the armed receptacles. NP: Nosepoke

**Experiment 1. **An initial experiment was carried out in order to evaluate both the overall phenotype of the R6/2 mouse and the specific capacity of both wild-type (WT) and R6/2 mice to learn a simple alternation rule, requiring them to switch between two locations to retrieve water reinforcement.****R6/2 mice were tested weekly from 7 to 10 weeks old. The subjects were a cohort of 44 naïve female mice evenly split by genotype in four groups of 11 mice each. Each group was tested in one cage. ****

Animals were water deprived in their home cages for 16 h prior to each phase of testing in the PhenoCube^TM^ system. During this period and the rest of the experiment feed were available ad libitum. The initial 7-wk test was split into three sections (as illustrated in Figure 3), with an initial 6-h Habituation day to acclimate the mice to the cage, followed by a 30-h session on the Alternation protocol and then a separate 6-h session with the Reversal protocol. The remaining test weeks were split into two 30-h sessions (Figure 3), with the nosepoke task reversed at the start of each session relative to the previous session. Despite the regular test schedule, some R6/2 mice still appeared unable to maintain licking behavior on the Alternation protocol; non-licking animals were switched to the Habituation protocol and ultimately removed from the cage as necessary. The switch to Habituation protocol was implemented electronically based on the RFID chip without disturbing the group or changing the protocol for the other mice in the social environment. The number of mutant mice still licking on the Alternation procedure at the end of each week’s testing (at 7, 8, 9 and 10 weeks) were 20, 12, 6, and 4 mice respectively. Note that when mice were switched to the Habitation protocol, they frequently resumed licking, indicating that their failure to lick on Alternation does not simply reflect a motor deficit.

**R6/2 mice test procedure.**
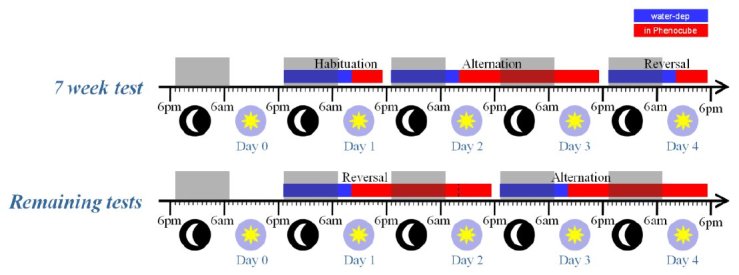

**Experiment 2. **BACHD mice were evaluated longitudinally, with testing at 16, 24, 36, and 52 weeks of age. The subjects were a cohort of 64 naïve female mice, split evenly into four cages of 8 mice per genotype (a total of eight cages), each tested in a single PhenoCube^TM^. In contrast to the R6/2 study, the animals were tested in a single long experimental session at each test age, with the procedures employed otherwise as described for the R6/2 experiment. The timeline for these test sessions is summarized in Figure 4, below. Note that, during the initial test at 16 weeks, the mice received an initial 24 hour period on the simple Habituation protocol, to allow them to become accustomed to the cage environment, before switching to Alternation for 48 hours. In the later tests, the mice were placed immediately onto the Alternation protocol, with the corner contingency reversing after 24 hours.

**BACHD mice test procedure.**
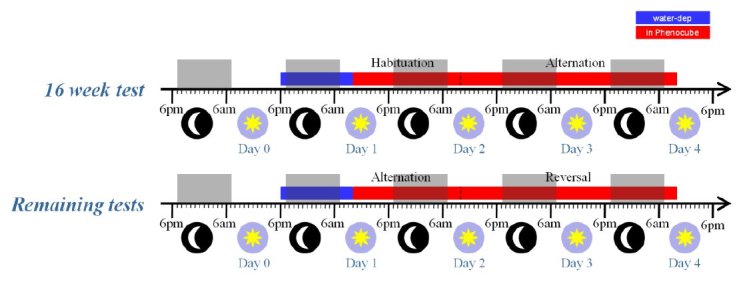

**Computer vision:**

In addition to data collected through the Intellicage hardware (visits to each of the four corners) general activity data was collected through PsychoGenics Inc’s proprietary computer vision automated video scoring system. Our computer vision detects and separates individual animals in the central area of the cage. As all mice in a cage had the same genotype we did not track their identity across the whole session time, although the system was developed since to do so; accordingly, these data are pooled across individuals within a cage. The primary measures currently implemented in this system are outlined below (note that animals within a corner are invisible to the camera and are accordingly not scored):

*Locomotion: *Measured as the distance travelled and normalized by the number of mice detected in each frame.

*Immobility:* Measured as the time when an individual mouse was detected not moving (maximum speed cutoff: 0.7 cm/s).

*Climbing:* Scored when a mouse was detected climbing on either of the climbing rods along the long wall of the cage or on the climbing structure (see Figure 1, left panel).

*Rearing:* Scored when an individual mouse was detected rearing against the walls of the apparatus.

**Data analysis:**

We analyzed data with mixed-design three-way ANOVA with SAS (SAS Institute Inc.) using Mixed Effect Models, based on likelihood estimation. The models were fitted using the procedure PROC MIXED using genotype as the between-subjects variable and light cycle and age as within-subjects variable. Significant interactions were followed by the simple effects analysis. Where multiple interactions are present involving a given factor, simple effects analyses were conducted only for the highest order interaction. An effect was considered significant if *p* < 0.05.

## Results

**Experiment 1. Analysis of R6/2 mice**.

*Corner Entries: *R6/2 and WT mice did not exhibit differential frequency of corner entries. Both groups visited the corners equally often, showed more visits during the dark light cycle, and reduced corner entries with repeated testing (Figure 5A; Table I; Age and Cycle main effects: *p*s < 0.05; simple effects analysis: *ps < *0*.*0001). R6/2 mice showed slightly larger differences across the day cycle although the differences between groups were not significant within each phase (Cycle x Genotype interaction: *p *< 0.05; simple effects analysis: *p*s > 0.11).

*Licking:* Analysis of licking behavior pointed at increased thirst in R6/2 mice during the dark period. R6/2 mice licked more frequently than the WT controls during the dark phase, although the effect was not significant at 10 weeks of age. Surprisingly the WT licked as frequently during the light as during the dark cycle (Figure 5B; Table 1; Genotype x Age x Cycle interaction: *p *< 0.01; simple main effects: *p*s < 0.05).

*Repeats:* R6/2 mice re-entered visited corners more frequently than the WT controls, at all ages, which reflects the perseverative responding R6/2 mouse behavior, which was (although to a lesser extent) also manifested in terms of their alternation performance (see below). This effect appeared more pronounced during the light period (Figure 5C; Table 1; Genotype x Cycle interaction: *p *< 0.01; simple main effects: *p*s < 0.05).

*Alternations:* As mentioned above, perseverative responding R6/2 behavior was also apparent in the spatial alternation performance; however genotypic differences were not as robust at older ages when compared to the deficits detected in repeated behavior. R6/2 animals alternated less than WT mice between active corners, where water could be obtained, although the differences were not significant at 9 weeks or at 10 weeks during the dark phase. Whereas WT mice alternated to the same extent during the light and dark cycle, R6/2 mice showed a further decrease in alternation during the light cycle phase at 8 weeks of age (Figure 5D; Table 1; Genotype x Age x Cycle interaction: p < 0.05; Simple Main Effects: *p*s < 0.05).

*Collected Rewards:* Despite the behavioral differences reported above R6/2 and WT mice had comparable levels of access to water reinforcement earned through the experimental contingencies. The proportion of entries to an active corner in which mice nosepoke and, likely, accessed the water reinforcement was similar between groups (Figure 5E, Table 1). There was a marginal non significant increase in collected rewards with repeated testing (Age main effect*: p* < 0.1).

**Table 1. Summary of the main effects and interaction following statistical analysis for the behavioral measures for Experiment 1 (R6/2).** Note: *: p<.05, **: p<.01, #: p<.001, NS: p>.05

Behavior	R6/2					
	Genotype	Age	Cycle	G x A	G x C	G x A x C
	(G)	(A)	(C)			
	F(1,38)	F(3,56)	F(1,38)	F(3,56)	F(1,38)	F(6,66)
**Corner Entries**	NS	4.13*	96.72#	NS	4.19*	NS
**Licking**	17.46#	3.67*	149.36#	5.26**	140.32#	4.14**
**Repeats**	83.97#	NS	NS	NS	21.80#	NS
**Alternations**	33.25#	NS	5.66*	NS	7.30*	2.39*
**Collected Rewards**	NS	NS	NS	NS	NS	NS

**R6/2 behavioral measures as a function of genotype, age, and light cycle.**
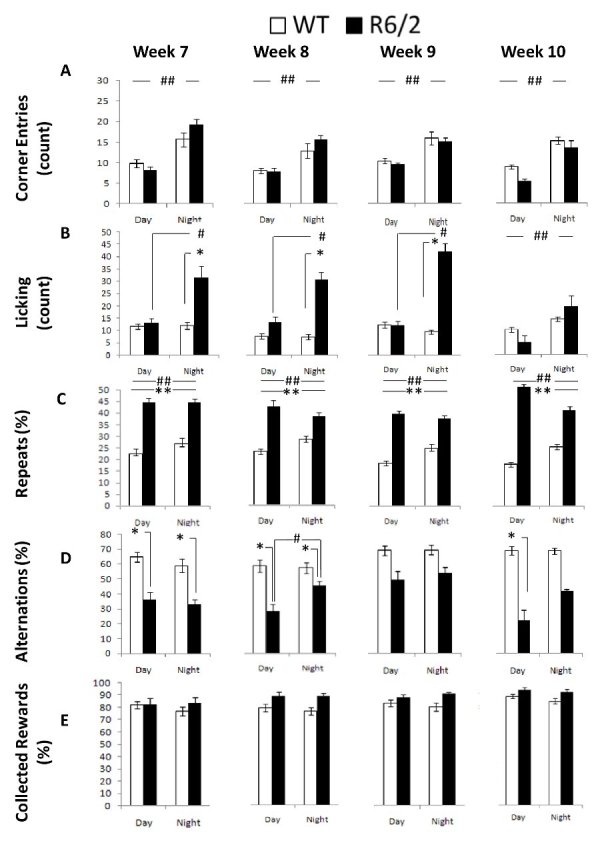
*: significant genotype differences within each light phase, at each age; **: significant genotype effect for each age, independent of cycle; #: significant differences due to light cycle for each genotype and age; ##: significant differences due to light cycle for each age independently of genotype.

**Table 2. Summary of statistical results for the computer vision measures for Experiment 1 (R6/2).** Notes: *: p< 0.05, **: p< 0.01, #: p< 0.001, NS: p>.05

Behavior	R6/2					
	Genotype	Age	Cycle	G x A	G x C	G x A x C
	(G)	(A)	(C)			
	F(1,2)	F(3,6)	F(1,2)	F(3,6)	F(1,2)	F(6,6)
**Locomotion**	NS	NS	136.16#	NS	NS	NS
**Time Immobile**	205.80#	6.91*	28.4*	NS	NS	NS
**Rearing/Climbing**	45.53*	NS	NS	NS	NS	NS

Consistent with earlier findings 9 ^,^ 12, these computer vision-based measures overall suggested lower locomotor activity and decreased motor function of R6/2 mice compared to WT mice. Details of these results are summarized below and in Table 2.

*Locomotion*. This group measure, obtained from the computer vision system, showed an overall increase in the activity of all mice during the night independently of genotype (Figure 6A; Table 2, Cycle main effect: *p* < 0.001). R6/2 mice showed a slight but not significant decrease in locomotion.

*Time Immobile*. From the computer vision it was also clear that R6/2 mice were more immobile over the whole testing period. As expected both groups were less immobile during the dark phase, while all animals became slightly more immobile with repeated testing (Figure 6B; Table 2, Genotype, Age and Cycle main effects: *p* < 0.001).

*Rearing/Climbing*. R6/2 mice reared and climbed less (Figure 6C; Table 2, Genotype main effect: *p* < 0.05). Although rearing seemed higher during the night there were no significant effects of either cycle or age.

**Behavioral measures derived from the computer vision of Experiment 1 (R6/2) as a function of genotype, age, and light cycle.**
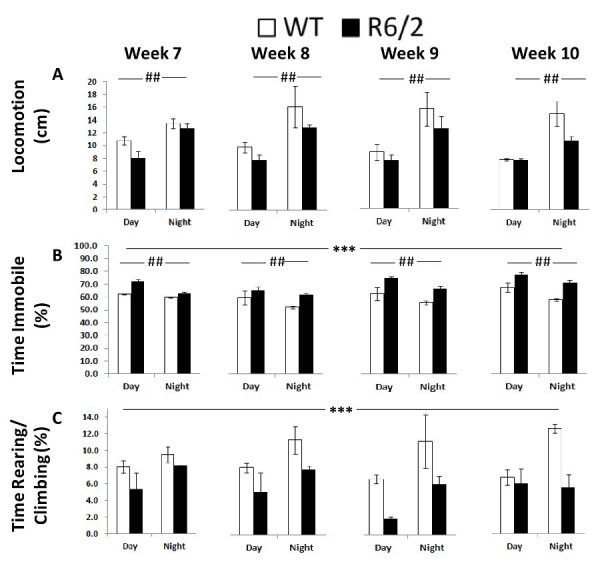
***: significant genotype effect, independent of cycle or age; ##: significant differences due to light cycle for each age independently of genotype.

**Experiment 2. Analysis of BACHD mice:**

*Corner Entries:* BACHD mice exhibited a different phenotype than R6/2 mice in terms of the degree of corner visits: R6/2 mice did not differ from their WT controls in terms of the frequency of corner visits however, BACHD mice entered the corners significantly less than WT mice at all ages and cycle phases with the exception of the dark phase at 16 weeks of age (Figure 7A; Table 3; Genotype x Age x Cycle interaction: *p*< 001; simple main effects: *p*s < 0.05). Both groups were more active during the dark period (Cycle main effect: *p*< 0.001).

*Licking:* Similar to the phenotype of R6/2 mice, BACHD mice exhibited increased thirst, which was not detected at the older ages (i.e., 10 weeks of age for R6/2 mice and 52 weeks of age for BACHD mice). At earlier ages, the BACHD mice licked more than did WT mice irrespective of cycle phase, but this difference waned as testing progressed (Figure 7B; Table 3; Genotype x Age x Cycle interaction: *p* < 0.01; simple main effects: *p*s < 0.05). As expected, both groups licked more during the night period (with the exemption of WT mice at 36 weeks of age) and, unexpectedly, licked less as testing progressed (Age main effect: *p* < 0.001).

*Repeat visits:* Perseverative responding was apparent only during early test ages for BACHD mice. BACHD mice re-entered previously visited corners more frequently than did WT controls at 16 and 24 weeks of age but less at 52 weeks of age (Figure 7C; Table 3; Genotype x Age interaction: *p* < 0.001; simple main effects: *p*s < 0.05). Both groups made more repeats when they were active during the dark phase and made fewer repeats as testing continued (Age and Cycle main effect: *p*s < 0.001).

*Alternation. *Similar age-dependent patterns of genotypic differences were also observed with spatial alternation performance. BACHD mice alternated less than WT during both cycle phases at 16 weeks of age but the effect was not apparent at later ages. BACHD mice alternated more during the light phase at all ages, while this difference was only observed in WT controls at 16 weeks of age. (Figure 7D; Table 3; Genotype x Age x Cycle interaction: *p* < 0.001).

*Rewards collected: *Interestingly, BACHD mice exhibited higher efficiency of reward collection than WT animals, a characteristic that became more pronounced with age. For instance, starting at 24 weeks of age BACHD mice were more likely than WT mice to nosepoke in active corners. Both groups, surprisingly, were slightly but consistently less likely to collect rewards during the dark phase, particularly the BACHD mice (Figure 7E; Table 3; Genotype x Age and Genotype x Cycle interactions: *p*s < 0.05; simple main effect: *p*s < 0.05).

**Table 3. Summary of the statistical analyses for individual behavioral measures for Experiment 2 (BACHD).** Notes: *: p< 0.05, **: p< 0.01, #: p< 0.001, NS: p>.05

Behavior	BACHD					
	Genotype	Age	Cycle	G x A	G x C	G x A x C
	(G)	(A)	(C)			
	F(1,57)	F(3,160)	F(1,57)	F(3,160)	F(1,57)	F(6,160)
**Corner Entries**	42.55#	47.93#	480.78#	7.05#	6.13*	12.19#
**Licking**	17.24#	51.31#	168.08#	3.50*	12.62#	3.39**
**Repeats**	NS	52.81#	158.69#	12.04#	NS	NS
**Alternations**	NS	6.70#	80.24#	5.07**	NS	9.01#
**Collected Rewards**	43.59#	32.99#	31.71#	17.75#	4.57*	NS

**BACHD behavioral measures as a function of genotype, age, and light cycle.**
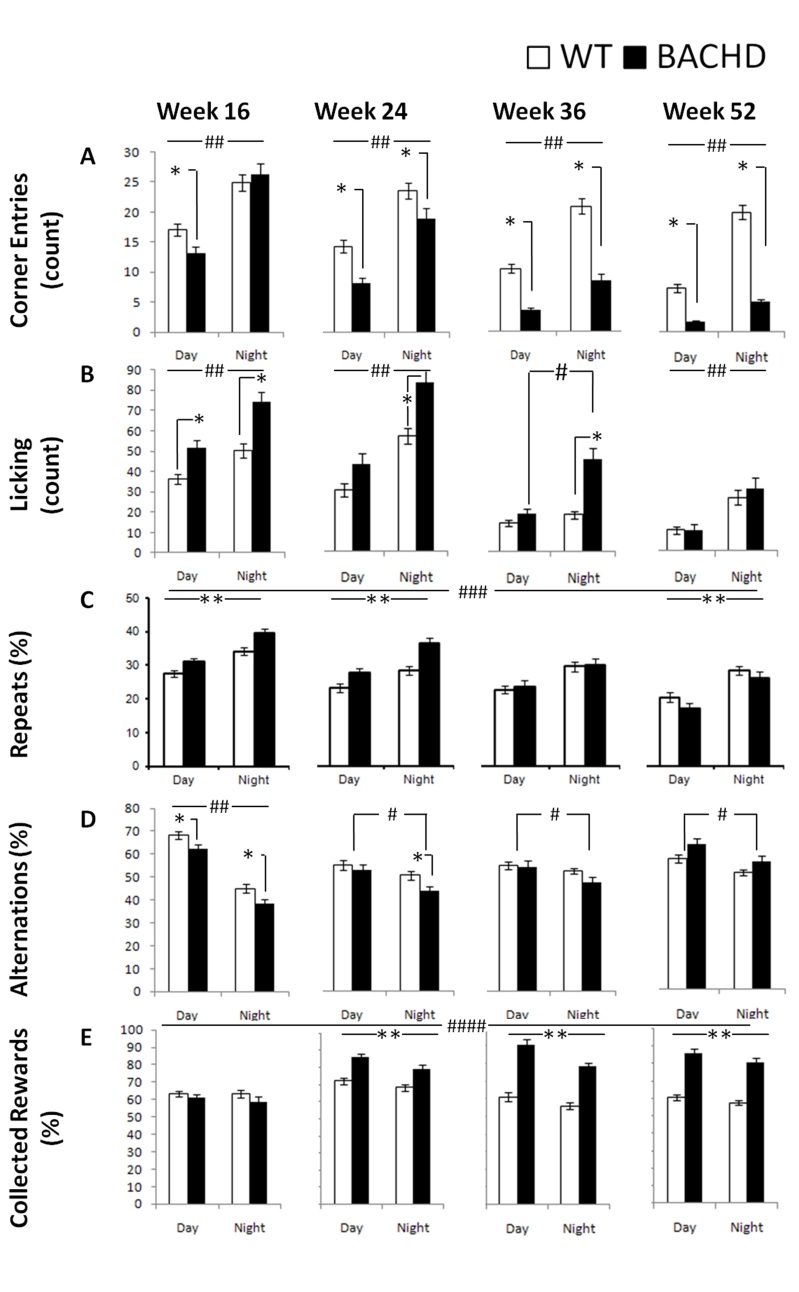
*: significant genotype effect for each age within each cycle; **: significant genotype effect for each age, independent of cycle; #: significant differences due to light cycle for each genotype and age; ##: significant differences due to light cycle for each age independently of genotype; ###: significant differences due to light cycle independently of genotype and age; ####: significant differences due to light cycle and genotype independently of age.

**Table 4. Summary of the statistical analyses for the computer vision derived measures for Experiment 2 (BACHD).** Notes: *: p< 0.05, **: p< 0.01, #: p< 0.001, NS: p>.05

Behavior	BACHD					
	Genotype	Age	Cycle	G x A	G x C	G x A x C
	(G)	(A)	(C)			
	F(1,6)	F(3,18)	F(1,6)	F(3,18)	F(1,6)	F(6,18)
**Locomotion**	149.53#	15.78#	127.02#	NS	NS	9.79#
**Immobility**	58.09#	6.62**	12.88*	NS	NS	NS
**Rearing Climbing**	7.22*	NS	18.82**	4.05*	NS	5.81**

Consistent with the R6/2 locomotor activity and motor function phenotype summarized above, BACHD mice exhibited lower locomotor activity, higher immobility, and lower rearing and climbing than WT mice. Some of these differences were more pronounced than in the R6/2 mice. Details of these results are summarized below.

*Locomotion: *BACHD mice were hypoactive relative to WT control mice at all ages and during both cycle phases. Activity was higher for both groups during the dark phase at 16, 24, and 36 weeks, as expected, but not at 52 weeks (Figure 8A, Table 4; Genotype x Age x Cycle: *p*< 0.001; simple main effects: *ps < *0*.*05).

*Time Immobile:* BACHD mice spent more time immobile than WT mice at all ages and light phases (Figure 8B, Table 4, Genotype main effect: *p* < 0.001). Both groups spent more time immobile during the light phase and slightly more as they aged (Cycle and Age main effects: *p*s < 0.02).

*Rearing & Climbing:* BACHD mice reared and climbed less than WT mice at 16 and 52 weeks of age. WT reared and climbed more during the dark at 16 and 36 ages but less at 52 weeks, whereas BACHD only climbed more at 36 weeks of age (Figure 8C, Table 4, Genotype x Age x Cycle: *p *< 0.01; simple main effects: *p*s < .05

**BACHD behavioral measures derived from the computer vision as a function of genotype, age, and light cycle:**
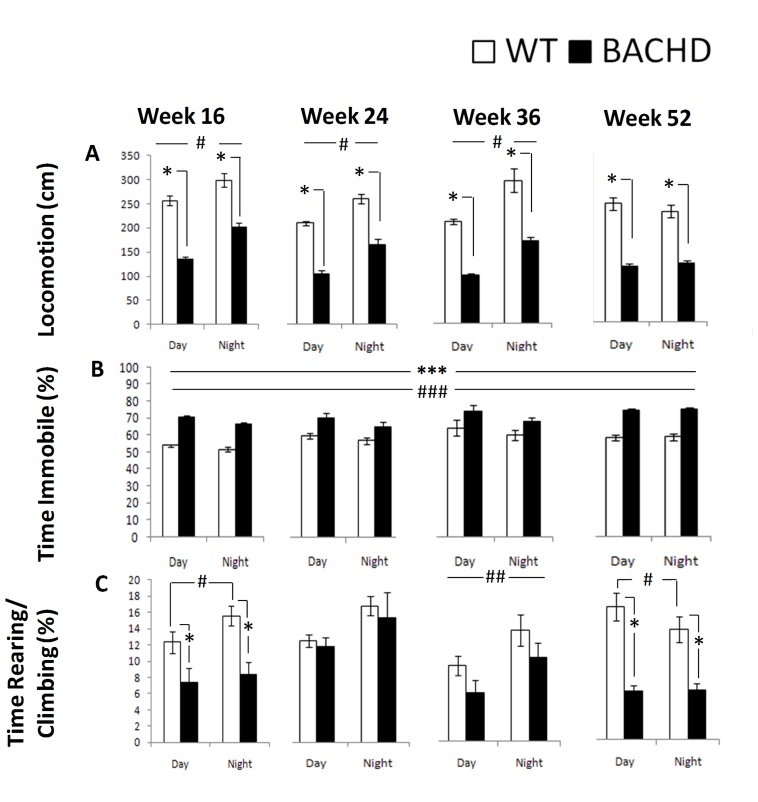
*: significant genotype effect for each age within each cycle; ***: significant genotype effect independent of cycle and age; #: significant differences due to light cycle for each genotype and age; ##: significant differences due to light cycle for each age independently of genotype; ###: significant differences due to light cycle independently of genotype and age.

## Discussion

Here we demonstrated the utility of our novel high-throughput phenotyping system, PhenoCube^TM^ by characterizing two different genetic mouse models of HD. Our observations reveal the sensitivity and efficiency of this system in capturing genotypic differences and tracking the course of the disease in multiple behavioral domains. Specifically, mice tested on this platform exhibited robust differences across genotypes in cognitive performance, motor behavior, and circadian patterns (circadian data are described separately in 14). These differences were captured by a fully automated system over a (user-specified) wide time window and age range using electronic behavioral actuators (chip reader and antenna from Intellicage) as well as computer vision tools.

Rudenko et al 25 assessed spatial alternation performance of R6/2 mice from the age of 8 weeks, primarily via patrolling task where the position of the correct corner was changed in a clock-wise manner following a visit to a correct corner and at least one nose-poke in that corner. They observed that R6/2 mice made significantly more place errors than did WT mice. Their findings parallel the decreased proportion of alternation and increased proportion of repeat visits detected as early as 7 weeks of age in our study. Importantly, perseverative responding has also been observed in HD patients as assessed in different tasks 26 ^,^ 27 ^,^ 28 ^,^ 29.

Relative to the R6/2 mouse, the BACHD model is not so well-characterized in terms of associated cognitive deficits, although they have been shown to have normal response acquisition in an operant task and 30 and motivational deficits in a progressive ratio task 31 ^,^ 20 stated that, in a procedural task, BACHD mice showed a deficit during reversal but not acquisition. The performance of the control mice in their study, however, was rather variable being close to chance levels at the end of both training and testing. In our unpublished studies we have seen both deficits during acquisition and reversal in a 2 choice swim task, but the results were neither consistent nor robust, suggesting that a possible deficit could be uncovered with a task that elicits more stable performance in both the transgenic and control groups. While the BACHD mice in this study, like the R6/2 mice, initially did appear to show stable tendency to make more repeat visits than controls, this phenotype declined with increasing age, with significant increases in repeat visits seen only at 16 and 24 weeks, with a reduced rate of repeat visiting detected at 52 weeks, while the R6/2 phenotype remained stable with increasing age.

The R6/2 mice also revealed an increase in drinking behavior relative to WT controls during the dark phase between 7-9 weeks of age, whereas there were no significant differences during the light cycle. This result is also consistent with Rudenko et al 25, who observed significant differences in the frequency of licks between 7-9 weeks of age only during the active period (containing the entire dark phase and the first half of the light phase). Different from their results however, the genotypic difference in drinking behavior disappeared at 10 weeks of age in our study essentially due to a decrease in the lick frequency of R6/2 mice, particularly during the dark phase. Note that IntelliCage does not measure the amount of liquid consumption but rather the number and duration of licking behavior. Thus, we used the number of licks as a proxy for amount of water consumed by mice (see also 25).

There were no differences between genotypes in terms of the total number of corner visits at least during their test ages (i.e., 7-10 weeks of age). This result is also consistent with Rudenko et al 25 who observed significant decreases in corner visit frequency starting from 11-12 weeks of age. Efficiency in collecting rewards (operationalized as nose poking in correct corner visits) was equivalent in WT and R6/2 mice. The rate of such trials suggests that irrespective of genotype, our mouse subjects had a weak tendency to explore when in the correct corners. Overall, we had the opportunity to compare our results with previous findings (i.e., 25) in the case of R6/2 mice, which revealed a high degree of correspondence between studies.

BACHD animals exhibited significantly reduced numbers of corner visits but significant increases in their tendency to make water seeking nosepoke responses when entering a correct corner compared to WT controls. Note that this increase in efficiency of reward collection does not necessarily indicate enhanced cognitive performance of BACHD mice but rather the suppression of alternative behaviors such as exploration and curiosity (see also 25), since the BACHD animals may only visit corners in order to retrieve water. In addition, our study using the progressive ration task, suggests that incentive motivation (which energizes behavior in order to obtain reward) is deficient in the BACHD mice 31.

The BACHD mice exhibited increases in drinking behavior relative to WT controls, though this phenotype disappeared at the final age of testing (52 weeks of age) due to a decrease in the lick frequency of BACHD mice, particularly during the dark phase. These findings regarding drinking behavior are consistent with previously reported results from the R6/2 mice 25 ^,^ 32. Increased thirst has also been shown to characterize the HD progression in the clinic 32. Importantly, perseverative responding has also been observed in HD patients as assessed in different tasks 26 ^,^ 27 ^,^ 28 ^,^ 29.

We also evaluated the motor behavior of the R6/2 and BACHD mice using the computer vision software revealing that maybe R6/2 but clearly BACHD mice had shorter total path length, (not significant for R6/2 mice), reduced levels of rearing and climbing behavior, and higher immobility time. These results are consistent with previous reports of decreased locomotion 9 ^,^ 33, rearing-climbing 9, and imbalance and motor coordination problems 9 ^,^ 20 in R6/2 and BACHD mice. The performance of R6/2 mice particularly paralleled the results of Rudenko et al 25 who tested these mice on open field 9 ^,^ 12. They observed significant decrease in number of rearings as early as 6 weeks of age, however differences in distance traveled occurred reliably at 12 weeks of age. Light cycle was found to affect many of our computer vision measures, which has also been reported in studies that tested WT mice in open field (e.g., 34 ^,^ 35 at least for C57BL/6).

Although the differences in locomotion were in the expected direction, these differences were not statistically significant for the R6/2 mice (*p*=.31). On the other hand, same R6/2 mice were significantly more immobile than their wild-type controls. The dissociation between these two measures is possible since immobility and locomotion (i.e., path length) are not perfectly complementary given all other alternative behaviors animals engage in (e.g., grooming). A more detailed characterization of behavior via computer vision system in future studies can clarify the dynamics that might underlie this dissociation.

Since our computer vision application does not track the individual animal identities in a consistent manner (see below for details), in order to compare genotypes in terms of computer vision indices, mice with the same genotype were housed in the same cage. While, we could track the identity of a given mouse prior to and following corner exits (based on time-stamped transponder detections), these periods of identity tracking were constrained by instances of occlusions, where “identity” swaps are likely. Ongoing development of the computer vision application is focused on reliable individual tracking and social behavior. Another advantage of genotype-based housing in our specific case was to avoid possible dominance of the WT mice over R6/2 and BACHD mice minimizing their access to resources. This would have constituted an important confound during behavioral phenotyping of these mice. Certain behavioral phenotypes of R6/2 and BACHD mice (e.g., disrupted sleep patterns) could also directly interact with WT mouse behavior. Similar factors however could also constitute a disadvantage of genotype-based housing schemes as they could inflate the degree of behavioral phenotype. For instance, disrupted sleep pattern of a given mouse could also disrupt the sleep patterns of other mice in the same cage.

Group housing mice during testing might itself exert some disadvantages. One of the likely problems with this approach is that group housing might induce social ranking dynamics, which might in turn influence behavior and task-related performances. In this study, we did not specifically evaluate social ranking and thus could not capture these possible relations. On the other hand, in combination with marking animals for individuation, analytical tools can be developed to evaluate social behavioral dynamics based on computer vision application endpoints. For instance, huddling behavior and/or dispersal of animals in the home-cage might provide important data regarding social behavior. Genotype-based housing or group-housing itself exerts a trade-off that needs to be evaluated given what is known about the mouse lines to be tested.

Another important issue is the number of subjects to test in a given case. In our case, the number of subjects was chosen to maximize the sample size (as allowed by regulations). Power analysis shows that group sizes of 3-11 mice for most of the parameters measured in the R6/2 mice at 8 weeks of age (namely: corner entries, licking count, percentage of repeats, percentage of alternations, percent time immobile and percent time rearing-climbing) are sufficient to detect a 50% improvement of a potential therapy (final number depends on the parameter measured; α= 0.05, 80% power). A similar sample size allows the evaluation of potential therapies at 10 weeks of age R6/2 mice in the PhenoCube^TM^ system when measuring percent alternations, percent time immobile and percent time rearing-climbing. In the BACHD mice, power analysis indicates that group sizes of 4-16 mice are sufficient to detect a 50% effect improvement at 36 weeks of age when measuring corner entries, collected rewards, and locomotion. If the effect of the potential therapy is examined at 52 weeks of age, group sizes of 4-9 mice are needed when measuring corner entries, collected rewards, locomotion, percent time immobile and percent time rearing-climbing.

One of the important features of the data gathered from this study is the increased licking that could be interpreted as increased thirst of both R6/2 and BACHD mice coupled with the use of water as reward. Increased licking phenotype might constitute a confound for behavioral tasks that set the rules for access to water reward 32. That have been said, the canonical interpretation of higher thirst would be increased motivation for water reward, which if anything would be expected to lead to more well-structured (and possibly less disrupted) goal-directed behaviors. On the other hand, several phenotypes such as the frequency of corner visits might have been masked by higher motivation for water reward at least in the R6/2 mice.

Note that the aim of this study was not only to characterize the disease profiles of two different mutations, but instead to demonstrate the utility of the PhenoCube^TM^ in mouse models of HD. Further work can test different mouse models at the same age (e.g., BACHD vs. R6/2 mice at 8 weeks of age) with identical experimental protocols to enable a direct comparison of mouse models of HD using PhenoCube^TM^ as a tool. Another interesting possibility for future studies would be mixing different genotypes in a cage and comparing their performance to conditions where mice of the same genotype are housed together (as in this study). This would enable the differentiation of disease progression in the presence and absence of mice with HD and might reveal possibly differential social behavior dynamics compared to genotype-based housing scheme. Finally, defining the accuracy of nose-poke based on randomly signaled water recesses would constitute a test of cued discrimination performance, which can be compared to the spatial discrimination of recesses as in the case of this study. These two different strategies are known to rely on different mechanisms (e.g., Ciamei & Morton, 2009).

Overall, these results in HD mouse models characterize PhenoCube^TM^ as an ecologically valid, sensitive, and efficient system in detecting and tracking the course of the phenotype progression concurrently across multiple behavioral measures. As practice effects are possible in longitudinal studies, confirmation of disease progression should be obtained using cross-sectional designs. Importantly, PhenoCube^TM^ allows assessment of behavior in non-stressful conditions and of grouped mice without any handling of the animals. Further refinement of the computer vision for assessment of social behavior will allow the study of complex interactions in heterogeneous groups of subjects, a topic of particular interest in preclinical work with models of schizophrenia and autism. In addition, the automated computer vision scoring provides measures of behavior 30 times/s, 24 h/day, for up to 6 consecutive days, giving unprecedented access to the secret life of mice (and rats in the near future), and offering the potential to discover unexpected aspects of their individual and group behavior. Critically, the ability to conduct longitudinal studies is necessary for not only validation of disease models but also for the initial screening of potential symptomatic treatments and therapeutic interventions. Results gathered from PhenoCube^TM^ can be used to guide further efforts using traditional assays. Thus, this new platform can be beneficial in eliminating the bottleneck in target validation and drug discovery.

## References

[1] Maggi S, Luciana G, Heise I, Nieus T, Balcı F, Wells S, Tocchini-Valentini GP, Mandillo S, Nolan PM, Tucci V. A cross-laboratory investigation of timing endophenotypes in mouse behavior. Timing & Time Perception. in press.

[2] Lipp, H.-P. (2005). High-throughput and Automated Behavioural Screening of Normal and Genetically Modified Mice, Business Briefing: Future Drug Discovery.

[3] Gallistel CR, King AP, Daniel AM, Freestone D, Papachristos EB, Balci F, Kheifets A, Zhang J, Su X, Schiff G, Kourtev H. Screening for Learning and Memory Mutations: A New Approach. Xin Li Xue Bao. 2010 Jan 30;42(1):138-158. PubMed PMID:20352069. 20352069 10.3724/SP.J.1041.2010.00138 PMC2844986

[4] Gray M, Shirasaki DI, Cepeda C, André VM, Wilburn B, Lu XH, Tao J, Yamazaki I, Li SH, Sun YE, Li XJ, Levine MS, Yang XW. Full-length human mutant huntingtin with a stable polyglutamine repeat can elicit progressive and selective neuropathogenesis in BACHD mice. J Neurosci. 2008 Jun 11;28(24):6182-95. PubMed PMID:18550760. 18550760 10.1523/JNEUROSCI.0857-08.2008 PMC2630800

[5] Mangiarini L, Sathasivam K, Mahal A, Mott R, Seller M, Bates GP. Instability of highly expanded CAG repeats in mice transgenic for the Huntington's disease mutation. Nat Genet. 1997 Feb;15(2):197-200. PubMed PMID:9020849. 9020849 10.1038/ng0297-197

[6] Ciamei A, Morton AJ. Rigidity in social and emotional memory in the R6/2 mouse model of Huntington's disease. Neurobiol Learn Mem. 2008 May;89(4):533-44. PubMed PMID:18069020. 18069020 10.1016/j.nlm.2007.10.009

[7] Ciamei A, Morton AJ. Rigidity in social and emotional memory in the R6/2 mouse model of Huntington's disease. Neurobiol Learn Mem. 2008 May;89(4):533-44. PubMed PMID:18069020. 18069020 10.1016/j.nlm.2007.10.009

[8] Lione LA, Carter RJ, Hunt MJ, Bates GP, Morton AJ, Dunnett SB. Selective discrimination learning impairments in mice expressing the human Huntington's disease mutation. J Neurosci. 1999 Dec 1;19(23):10428-37. PubMed PMID:10575040. 10575040 10.1523/JNEUROSCI.19-23-10428.1999 PMC6782405

[9] Menalled L, El-Khodor BF, Patry M, Suárez-Fariñas M, Orenstein SJ, Zahasky B, Leahy C, Wheeler V, Yang XW, MacDonald M, Morton AJ, Bates G, Leeds J, Park L, Howland D, Signer E, Tobin A, Brunner D. Systematic behavioral evaluation of Huntington's disease transgenic and knock-in mouse models. Neurobiol Dis. 2009 Sep;35(3):319-36. PubMed PMID:19464370. 19464370 10.1016/j.nbd.2009.05.007 PMC2728344

[10] Morton AJ, Skillings E, Bussey TJ, Saksida LM. Measuring cognitive deficits in disabled mice using an automated interactive touchscreen system. Nat Methods. 2006 Oct;3(10):767. PubMed PMID:16990806. 16990806 10.1038/nmeth1006-767

[11] Pallier PN, Maywood ES, Zheng Z, Chesham JE, Inyushkin AN, Dyball R, Hastings MH, Morton AJ. Pharmacological imposition of sleep slows cognitive decline and reverses dysregulation of circadian gene expression in a transgenic mouse model of Huntington's disease. J Neurosci. 2007 Jul 18;27(29):7869-78. PubMed PMID:17634381. 17634381 10.1523/JNEUROSCI.0649-07.2007 PMC6672877

[12] Carter RJ, Lione LA, Humby T, Mangiarini L, Mahal A, Bates GP, Dunnett SB, Morton AJ. Characterization of progressive motor deficits in mice transgenic for the human Huntington's disease mutation. J Neurosci. 1999 Apr 15;19(8):3248-57. PubMed PMID:10191337. 10191337 10.1523/JNEUROSCI.19-08-03248.1999 PMC6782264

[13] Morton AJ, Wood NI, Hastings MH, Hurelbrink C, Barker RA, Maywood ES. Disintegration of the sleep-wake cycle and circadian timing in Huntington's disease. J Neurosci. 2005 Jan 5;25(1):157-63. PubMed PMID:15634777. 15634777 10.1523/JNEUROSCI.3842-04.2005 PMC6725210

[14] Oakeshott S, Port RG, Cummins-Sutphen J, Watson-Johnson J, Ramboz S, Park L, Howland D, Brunner D. HD mouse models reveal clear deficits in learning to perform a simple instrumental response. PLoS Curr. 2011a Nov 30;3:RRN1282. PubMed PMID:22512000. 22512000 10.1371/currents.RRN1282 PMC3327146

[15] Balci F, Day M, Rooney A, Brunner D. Disrupted temporal control in the R6/2 mouse model of Huntington's disease. Behav Neurosci. 2009 Dec;123(6):1353-8. PubMed PMID:20001119. 20001119 10.1037/a0017650

[16] Grote HE, Bull ND, Howard ML, van Dellen A, Blakemore C, Bartlett PF, Hannan AJ. Cognitive disorders and neurogenesis deficits in Huntington's disease mice are rescued by fluoxetine. Eur J Neurosci. 2005 Oct;22(8):2081-8. PubMed PMID:16262645. 16262645 10.1111/j.1460-9568.2005.04365.x

[17] Murphy KP, Carter RJ, Lione LA, Mangiarini L, Mahal A, Bates GP, Dunnett SB, Morton AJ. Abnormal synaptic plasticity and impaired spatial cognition in mice transgenic for exon 1 of the human Huntington's disease mutation. J Neurosci. 2000 Jul 1;20(13):5115-23. PubMed PMID:10864968. 10864968 10.1523/JNEUROSCI.20-13-05115.2000 PMC6772265

[18] Cepeda C, Ariano MA, Calvert CR, Flores-Hernández J, Chandler SH, Leavitt BR, Hayden MR, Levine MS. NMDA receptor function in mouse models of Huntington disease. J Neurosci Res. 2001 Nov 15;66(4):525-39. PubMed PMID:11746372. 11746372 10.1002/jnr.1244

[19] Cepeda C, Hurst RS, Calvert CR, Hernández-Echeagaray E, Nguyen OK, Jocoy E, Christian LJ, Ariano MA, Levine MS. Transient and progressive electrophysiological alterations in the corticostriatal pathway in a mouse model of Huntington's disease. J Neurosci. 2003 Feb 1;23(3):961-9. PubMed PMID:12574425. 12574425 10.1523/JNEUROSCI.23-03-00961.2003 PMC6741903

[20] Abada YS, Schreiber R, Ellenbroek B. Motor, emotional and cognitive deficits in adult BACHD mice: a model for Huntington's disease. Behav Brain Res. 2013 Feb 1;238:243-51. PubMed PMID:23123142. 23123142 10.1016/j.bbr.2012.10.039

[21] Kudo T, Schroeder A, Loh DH, Kuljis D, Jordan MC, Roos KP, Colwell CS. Dysfunctions in circadian behavior and physiology in mouse models of Huntington's disease. Exp Neurol. 2011 Mar;228(1):80-90. PubMed PMID:21184755. 21184755 10.1016/j.expneurol.2010.12.011 PMC4346330

[22] Morton AJ, Lagan MA, Skepper JN, Dunnett SB. Progressive formation of inclusions in the striatum and hippocampus of mice transgenic for the human Huntington's disease mutation. J Neurocytol. 2000 Sep;29(9):679-702. PubMed PMID:11353291. 11353291 10.1023/a:1010887421592

[23] NRC (1996) Guide for the care and use of laboratory animals. National Academy, Washington, DC.

[24] Jacobs GH, Fenwick JC, Calderone JB, Deeb SS. Human cone pigment expressed in transgenic mice yields altered vision. J Neurosci. 1999 Apr 15;19(8):3258-65. PubMed PMID:10191338. 10191338 10.1523/JNEUROSCI.19-08-03258.1999 PMC6782287

[25] Rudenko O, Tkach V, Berezin V, Bock E. Detection of early behavioral markers of Huntington's disease in R6/2 mice employing an automated social home cage. Behav Brain Res. 2009 Nov 5;203(2):188-99. PubMed PMID:19410605. 19410605 10.1016/j.bbr.2009.04.034

[26] Bäckman L, Robins-Wahlin TB, Lundin A, Ginovart N, Farde L. Cognitive deficits in Huntington's disease are predicted by dopaminergic PET markers and brain volumes. Brain. 1997 Dec;120 ( Pt 12):2207-17. PubMed PMID:9448576. 9448576 10.1093/brain/120.12.2207

[27] Lange KW, Sahakian BJ, Quinn NP, Marsden CD, Robbins TW. Comparison of executive and visuospatial memory function in Huntington's disease and dementia of Alzheimer type matched for degree of dementia. J Neurol Neurosurg Psychiatry. 1995 May;58(5):598-606. PubMed PMID:7745410. 7745410 10.1136/jnnp.58.5.598 PMC1073493

[28] Lawrence AD, Sahakian BJ, Rogers RD, Hodge JR, Robbins TW. Discrimination, reversal, and shift learning in Huntington's disease: mechanisms of impaired response selection. Neuropsychologia. 1999 Nov;37(12):1359-74. PubMed PMID:10606011. 10606011 10.1016/s0028-3932(99)00035-4

[29] Oscar-Berman M, Zola-Morgan SM, Oberg RG, Bonner RT. Comparative neuropsychology and Korsakoff's syndrome. III--Delayed response, delayed alternation and DRL performance. Neuropsychologia. 1982;20(2):187-202. PubMed PMID:6211634. 6211634 10.1016/0028-3932(82)90009-4

[30] Oakeshott S, Port RG, Cummins-Sutphen J, Watson-Johnson J, Ramboz S, Park L, Howland D, Brunner D. HD mouse models reveal clear deficits in learning to perform a simple instrumental response. PLoS Curr. 2011 Nov 30;3:RRN1282. PubMed PMID:22512000. 22512000 10.1371/currents.RRN1282 PMC3327146

[31] Oakeshott S, Port R, Cummins-Sutphen J, Berger J, Watson-Johnson J, Ramboz S, Paterson N, Kwak S, Howland D, Brunner D. A mixed fixed ratio/progressive ratio procedure reveals an apathy phenotype in the BAC HD and the z_Q175 KI mouse models of Huntington’s disease. PLOS Currents Huntington Disease. 2012 Apr 25 [last modified 2012 Sep 4]. Edition 1. 10.1371/4f972cffe82c0. 10.1371/4f972cffe82c0 PMC3729251 23925262

[32] Wood NI, Goodman AO, van der Burg JM, Gazeau V, Brundin P, Björkqvist M, Petersén A, Tabrizi SJ, Barker RA, Morton AJ. Increased thirst and drinking in Huntington's disease and the R6/2 mouse. Brain Res Bull. 2008 May 15;76(1-2):70-9. PubMed PMID:18395613. 18395613 10.1016/j.brainresbull.2007.12.007

[33] Gray M, Shirasaki DI, Cepeda C, André VM, Wilburn B, Lu XH, Tao J, Yamazaki I, Li SH, Sun YE, Li XJ, Levine MS, Yang XW. Full-length human mutant huntingtin with a stable polyglutamine repeat can elicit progressive and selective neuropathogenesis in BACHD mice. J Neurosci. 2008 Jun 11;28(24):6182-95. PubMed PMID:18550760. 18550760 10.1523/JNEUROSCI.0857-08.2008 PMC2630800

[34] Hostetter, R. (1966). Time of day effects on learning and open field activity. Psychon. Sci., 5, 257-258.

[35] Valentinuzzi VS, Buxton OM, Chang AM, Scarbrough K, Ferrari EA, Takahashi JS, Turek FW. Locomotor response to an open field during C57BL/6J active and inactive phases: differences dependent on conditions of illumination. Physiol Behav. 2000 May;69(3):269-75. PubMed PMID:10869592. 10869592 10.1016/s0031-9384(00)00219-5

